# Diagnosis and treatment of hepatic hilar lymph node metastasis in hepatic alveolar echinococcosis patients: a real-world single-center experience

**DOI:** 10.3389/fonc.2025.1708936

**Published:** 2026-01-21

**Authors:** Fengyuan Tian, Yinshu Zhou, Jinpeng Wang, Pengcai Feng, Xiangqian Wang, Mingyan Ma, Chaoliang Shangguan, Haihong Zhu

**Affiliations:** 1 College of Clinical Medicine, Qinghai University, Xining, Qinghai, China; 2 General Surgery Department, Qinghai Provincial People’s Hospital, Xining, Qinghai, China; 3 College of Clinical Medicine, Jinan University, Guangzhou, China

**Keywords:** hepatic alveolar echinococcosis, long-term prognosis, lymph node metastasis, lymph node dissection, predictive model

## Abstract

**Background:**

In patients with hepatic alveolar echinococcosis (HAE), germinal cells of *Echinococcus multilocularis* (EM) might invade regional lymph nodes (LNs) causing LN metastasis. If infected LNs are not removed during surgery, the risk of postoperative recurrence significantly increases. We aimed to develop a preoperative prediction model for HAE hepatic hilar LN metastasis using clinical and imaging data, summarize our center’s surgical experience, and analyze the long-term prognosis.

**Materials and methods:**

We retrospectively reviewed patients with HAE who underwent radical hepatectomy combined with systematic hepatic hilar LN dissection and targeted LN resection (SHD-TR) at our center from January 2016 to April 2025. We collected patients’ clinical data, imaging characteristics, surgical outcomes, and recurrence rates. Univariate and multivariate analyses were conducted to identify independent predictive factors for HAE hepatic hilar LN metastasis to construct a prediction model. Receiver operating characteristic (ROC) curves, precision-recall (PR) curves, calibration curves, and decision curve analysis (DCA) were used to evaluate the predictive performance of the model.

**Results:**

The independent predictive factors included LN diameter, nodular calcification, and nonenhancement. Recurrent disease was seen in four patients without LN metastasis and two patients with LN metastasis. Kaplan-Meier curves revealed no significant difference in disease-free survival (DFS) between the two groups (*P* = 0.920). The classification performance of the training and validation sets was consistent, with an area under the curve (AUC) of 0.895 [95% CI: 0.827-0.963] and 0.817 [95% CI: 0.669-0.964], respectively, for the ROC curve and 0.81 [95% CI: 0.685-0.904] and 0.721 [95% CI: 0.625-0.974], respectively, for the PR curve.

**Conclusion:**

The prognosis of hepatic hilar LN metastasis is similar to that of HAE without metastasis. Radical hepatectomy combined with SHD-TR is safe and effective for HAE patients with LN metastasis. The prediction model based on LN diameter, nodular calcification, and nonenhancement has a strong and stable discrimination ability for HAE LN metastasis.

## 1 Introduction

Alveolar echinococcosis (AE) is a serious helminthic zoonosis caused by the larvae of *Echinococcus multilocularis* (EM). It is primarily prevalent in Central Asia, Eastern Europe, and the pastoral regions of western China, and China alone accounts for more than 90% of the global burden, with more than 16,000 cases annually (1). In these regions, definitive hosts such as foxes and dogs excrete feces containing EM eggs. Humans, as intermediate hosts, become infected through contact with contaminated food or water (2). Once ingested, the eggs hatch in the intestine and release larvae called oncospheres. Oncospheres penetrate the intestinal mucosa using small hooks, enter the portal venous system, and migrate through the bloodstream, primarily settling in the liver, where they cause hepatic alveolar echinococcosis (HAE) (3). The biological features of HAE are similar to those of malignant tumors, characterized by progressive growth within the liver through budding or infiltration (4). This leads to the invasion of adjacent tissues, potentially causing damage to the liver parenchyma and critical structures such as the intrahepatic vasculature and bile ducts (5). Consequently, severe complications such as liver failure and obstructive jaundice may ensue. Notably, the liver’s robust compensatory mechanisms often mask symptoms until HAE has reached an advanced stage. During the intermediate and advanced phases, the germinal layer cells of EM detach and drain into regional lymph nodes (LNs) via the deep and superficial lymphatic systems, leading to LN metastasis (6, 7). Lymph flow is slow in the hepatic hilum region, which represents the first stop in the hepatic lymphatic return pathway, making it a critical site for more than 70% of metastases to hepatic hilar LNs (8). Metastatic hepatic LNs can fuse and invade hepatic structures, resulting in complications such as portal vein cavernous transformation and portal hypertension. The risk of postoperative recurrence increases when the infected hepatic hilar LNs are not removed. Hence, precise diagnosis of LN metastasis and timely treatment are crucial for good prognosis.

Early diagnosis of HAE predominantly depends on imaging techniques such as ultrasound, CT, and MRI. Enlargement, sand-like calcifications, nodular calcification, hypointense foci, and nonenhancement are the characteristic features of HAE metastatic LNs on CT scans (6). Nonetheless, these methods exhibit low detection rates for LN metastasis in HAE, compounded by poor accuracy, resulting in frequent misjudgments (9). For example, reactive hyperplastic LNs caused by chronic inflammation may also present as enlargement and calcification, and early metastatic LNs may even appear normal on imaging (8). Unfortunately, few studies exist in this area at present. A more straightforward and precise method for diagnosing LNs affected by HAE is urgently needed.

The primary treatment of HAE is surgery and radical hepatectomy is considered the main curative approach (10, 11). However, for patients with HAE and hepatic hilar LN metastasis, the World Health Organization (WHO) lacks a consensus on accurate preoperative diagnosis, the routine necessity of hepatic hilar LN dissection, and the extent of such dissection. Previous research suggests that hepatic hilar LN dissection is crucial for preventing residual lesions and reducing recurrence risk (6). Notably, hepatic hilar LNs account for 80% of total liver lymph drainage, making them the primary site for LN metastases (12). Considering surgical risks and patient experiences, in the region outside the hepatic hilar, the decision to perform LN dissection or solely remove suspicious LNs is critical. Various institutions have adopted different strategies for potential metastatic LNs, including only removing suspicious LNs, performing only hepatic hilar LN dissection, and dissecting all regional LNs with suspected metastasis. However, no study has defined the optimal treatment for HAE combined with LN metastasis. Our center performs systematic hepatic hilar LN dissection and targeted LN resection (SHD-TR) for patients with suspected hepatic hilar LN metastasis. SHD-TR can be summarized as further exploration and resection of potential metastatic LNs (abnormal LNs detected on preoperative imaging or during surgery) in the liver drainage area and adjacent intra-abdominal organs following hepatic hilar LN dissection. This surgical approach, which aims to maximize the clearance of metastatic LNs while preserving healthy LNs, has never been reported in HAE.

The main purpose of this study was to develop a novel preoperative diagnostic model for assessing hepatic hilar LN metastasis in patients with HAE and to review the surgical outcomes and prognosis of 512 patients with HAE at our center over the past nine years.

## 2 Materials and methods

### 2.1 Patient selection and study design

This study was approved by the Ethics Committee of Qinghai Provincial People’s Hospital (No (2023).-277). Given that this was a retrospective study, informed consent was not needed. All methods were carried out in accordance with the Declaration of Helsinki and relevant guidelines and regulations.

**Figure 1:**
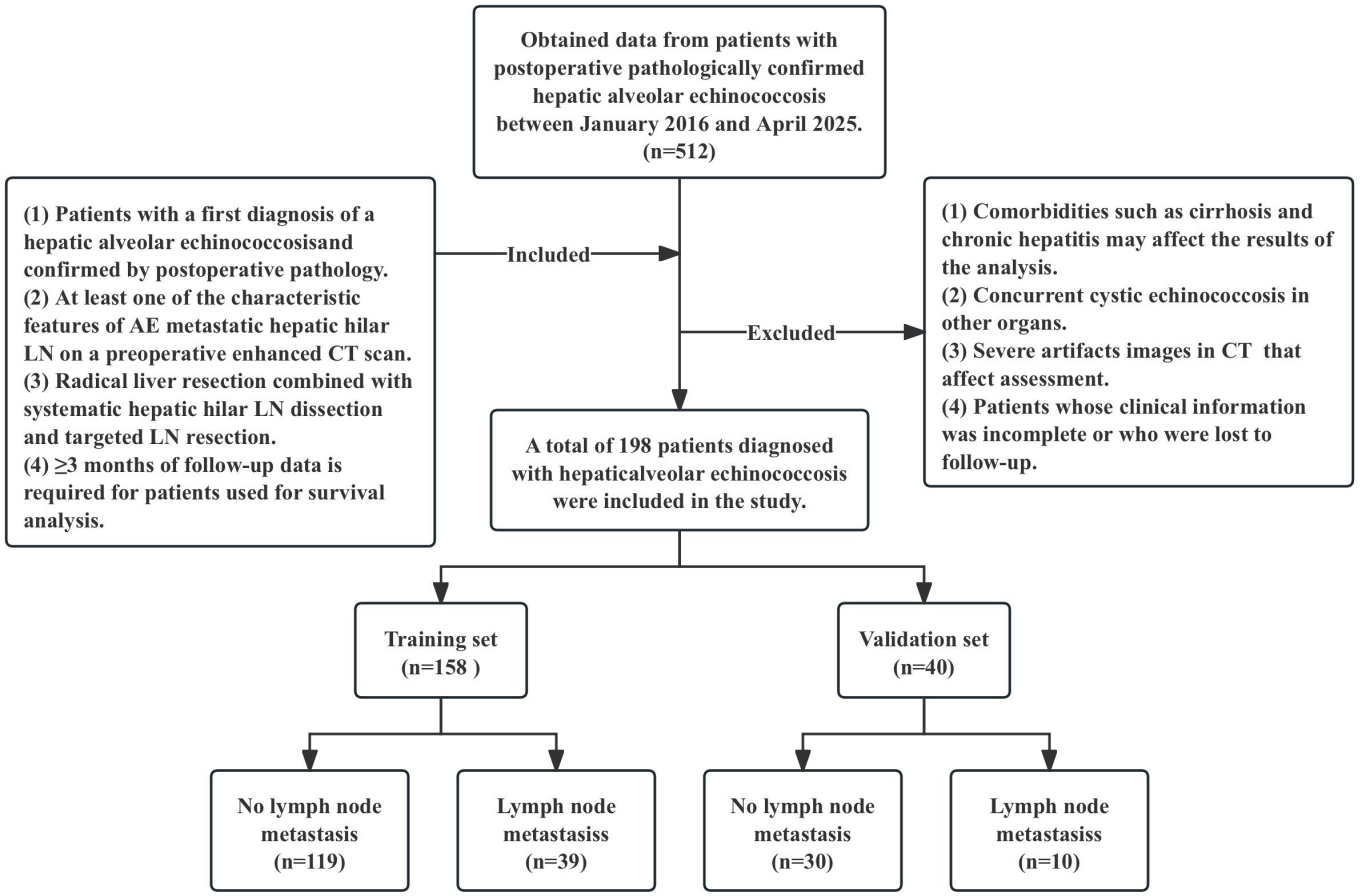
Flowchart illustrating selection criteria of patients in the training, and validation sets.

A comprehensive review and selection were conducted on 512 patients with pathologically confirmed HAE from January 2016 to April 2025. The inclusion criteria for patients were as follows (1): a diagnosis of HAE confirmed by postoperative pathology (2); at least one of the characteristic features of AE metastatic hepatic hilar LN [enlargement (diameter > 7 mm), sand-like calcifications, nodular calcifications, hypointense foci, or nonenhancement] on preoperative contrast-enhanced CT (3); radical hepatectomy combined with SHD-TR; and (4) survival follow-up data for three months or longer. The exclusion criteria were as follows (1): complications such as liver cirrhosis and chronic hepatitis that may affect the results of the analysis (2); concurrent cystic echinococcosis in other organs (3); severe artifacts on CT that affect the assessment; and (4) incomplete clinical information or loss to follow-up. After strict screening, 198 participants were included. Postoperative pathological results were used as the gold standard; there were 49 cases of LN metastasis and 149 cases of reactive LN hyperplasia in the hepatic hilar. Patients were randomly divided into a training set (N = 158) and a validation set (N = 40) at an 8:2 ratio. The flowchart in **Figure 1** shows the details of the patient selection process.

### 2.2 CT image acquisition and analysis

Qinghai Provincial People’s Hospital utilized the 64-slice spiral CT (GE Lightspeed VCT, GE Healthcare, USA) for patient scans. The scanning range extended from the diaphragm to the pubic symphysis. The parameters for enhanced abdominal scanning followed the 2016 “CT Examination Technology Expert Consensus”: 120 kV, automatic tube current modulation, helical scanning with a pitch of 0.98-1.37, tube rotation speed of 0.6-0.8 s/rotation, 64 x 0.625 mm detector width, and 300 mm x 350 mm field of view. Iodixanol 300 (300 mg/mL iodine concentration) was used as the contrast agent and was administered intravenously at 3.0 mL/s using a high-pressure injector. The dosage was 1.5 mL/kg body weight, followed by a 30 mL saline flush. Scans in the arterial and venous phases were conducted at 30 and 55 s after injection, respectively.

**Figure 2:**
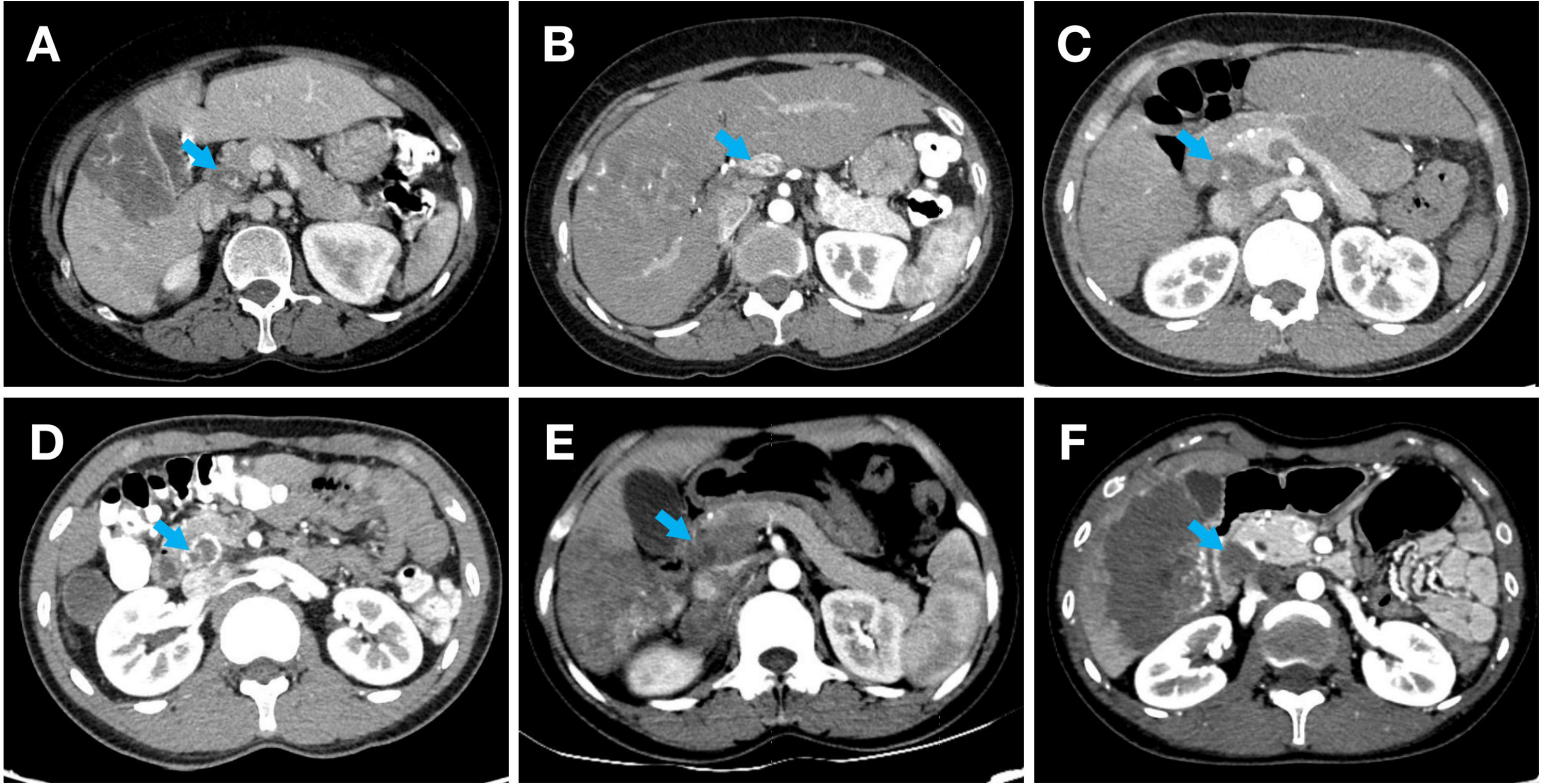
Contrast-enhanced CT scan features of metastatic LNs in HAE. **(A)** An enlarged LN with a diameter of 23 mm beside the portal vein, nodular calcification and low-density areas within the lesion, and heterogeneous enhancement on an enhanced scanning. **(B)** An enlarged LN with a diameter of 21 mm beside the common hepatic artery, sand-like calcifications within the lesion, and heterogeneous enhancement on enhanced scanning. **(C)** Enlarged and fused LNs with a diameter of approximately 39 mm behind the pancreatic head and in front of the inferior vena cava, nodular calcification within the lesion, and homogeneous enhancement on enhanced scanning. **(D)** Enlarged and fused LNs with a diameter of approximately 23 mm behind the pancreatic head and in front of the inferior vena cava, sand-like and eggshell-like calcifications around the lesion, and heterogeneous enhancement on enhanced scan. **(E)** Enlarged and fused LNs with a diameter of approximately 44 mm behind the pancreatic head and in front of the inferior vena cava, patchy slightly low-density shadows within the lesion, heterogeneous enhancement on enhanced scan, and patchy non-enhanced liquefactive necrotic areas within the lesion. **(F)** Enlarged and fused LNs with a diameter of approximately 23 mm behind the common bile duct, eggshell-like calcifications around the lesion, moderate enhancement at the edge of the lesion on enhanced scanning and no obvious enhancement within the lesion.

Two radiologists independently analyzed preoperative contrast-enhanced CT arterial phase images of patients with HAE using a double-blind method. In cases of discordant evaluations, a third radiologist intervened to review the images and establish a final consensus. The CT characteristics of potential metastatic LNs are illustrated in **Figure 2**. The imaging features collected in this study included the location of the hepatic lesion, hepatic lesion size, PNM stage, LN location, LN diameter, and number of LNs. To minimize differences and improve the credibility of the study results, the average values of the lesion and LN sizes measured by three radiologists were used for statistical analysis. In this study, the intraclass correlation coefficient (ICC) was used to assess the interobserver reliability of the imaging feature extraction method, with an ICC > 0.75 indicating good consistency.

### 2.3 Surgical procedure

Indications (1): Cardiopulmonary function can tolerate general anesthesia and surgery, and there are no other serious diseases before surgery (2). Liver function classified as Child-Pugh grade A-B (3). Preoperative imaging examinations or intraoperative explorations suggest the possibility of hepatic hilar LN metastasis (4). Patient consent to surgical treatment.

Contraindications (1): Patients with poor cardiopulmonary function who cannot tolerate general anesthesia and surgery (2). Child-Pugh class C (3). Patients who underwent cerebral hydatid surgery within one year (4). Patients with multiple pulmonary metastases (5). Patients who could not undergo radical surgery for other reasons.

Surgical procedure (1): An inverted L-shaped incision (25–30 cm) was made in the upper abdomen, and the abdomen was opened in layers (2). Abdominal exploration was performed after. Adhesions were released during exploration (3). Dissection of the liver: The round and falciform ligaments were transected with an ultrasonic scalpel up to the inferior vena cava area, and the second porta hepatis was dissected to expose the suprahepatic inferior vena cava (4). Dissection of the first porta hepatis: Glisson’s sheath was incised, and fatty and lymphatic tissues were removed to expose the common hepatic artery, proper hepatic artery, common hepatic duct, common bile duct, and portal vein. Each of these was suspended and marked with a rubber band, and an occlusive band was placed at the first porta hepatis (5). Sequential exploration and resection of potential metastatic LNs: The liver’s left lateral lobe was lifted to assess the gastric lesser curvature LNs. The left liver lobe was dissected by cutting and ligating the left triangular and coronary ligaments to assess the left subphrenic LNs. The lesser omental bursa was dissected to assess the celiac trunk LNs. A Kocher incision was used to dissect the inferior vena cava and renal vessels, exposing the inferior vena cava and abdominal aorta behind the pancreatic head to assess the corresponding LNs. With respect to the right liver lobe, the right triangular ligament was cut and ligated, the right coronary ligament was cut, and the right subphrenic LNs were assessed (6). Radical hepatectomy: The liver capsule was incised 1.0 cm from the edge of the lesion using electrocautery. The Pringle maneuver was used to occlude the hepatic hilum, according to the degree of intraoperative bleeding. The hepatic parenchyma was clamped and transected. The vascular and biliary structures near the proximal ends of the lesion were ligated with titanium clips, while the distal ends were ligated with 0 or 1 silk sutures and then transected. Bipolar electrocautery was used for hemostasis. If the lesion was large or fixed, which made it difficult to expose deep tissue or vessels and posing a risk of damaging critical vessels, a volume-reducing resection of the lesion could be performed first. Finally, the extent of invasion determined the need for repair and reconstruction of the diaphragm, inferior vena cava, hepatic veins, portal vein, or biliary tract. A schematic diagram of the surgical procedure for a 34-year-old female patient is shown in **Supplementary Figure 1**.

Postoperative management: Patients’ vital signs and liver function indicators were routinely monitored after surgery, with symptomatic relief and supportive treatment provided as needed. The Clavien-Dindo classification system was used to assess postoperative complications, and individualized interventions were used to assess complications.

Postoperative follow-up: Once liver function recovered, albendazole was routinely administered orally for two years to minimize the risk of occult lesions. To ensure medication adherence, document adverse drug reactions, assess postoperative complication prognosis, and evaluate long-term efficacy through imaging, our center recommends routine abdominal ultrasonography or CT at 1, 3, 6, 12, and 24 months post-surgery to monitor lesion recurrence and liver regeneration. Liver function tests are conducted to track recovery and assess the safety of albendazole. For patients with complex conditions, such as vascular reconstruction, PNM stage >2, or LN metastasis, we recommend lifelong annual follow-up.

### 2.4 Prognostic data analysis

Regular follow-up was carried out, with disease recurrence serving as the observation endpoint. Patient recurrence status and the duration from surgery to recurrence were documented to assess disease-free survival (DFS). Recurrence was categorized as margin recurrence, new liver lesions, LN recurrence, or distant organ metastasis. Survival outcomes were compared using Kaplan-Meier curves and log-rank tests. A Cox proportional hazards regression model was used to adjust for potential confounding factors.

### 2.5 Development and evaluation of the models

Using the training dataset, clinical and imaging parameters associated with hepatic hilar LN metastasis were analyzed through univariate and multivariate logistic regression to identify independent predictors of hepatic hilar LN metastasis (13). A prediction model was developed based on these independent predictors. The variance inflation factor (VIF) was calculated to assess multicollinearity among the variables, with a VIF greater than 10 indicating significant multicollinearity (14). The E value was calculated to assess the potential impact of unmeasured confounders on the independent predictors, thereby strengthening causal inference (15). A nomogram was constructed to visualize the prediction model. The area under the curve of the receiver operating characteristic (AUC-ROC) curve and precision-recall (AUC-PR) curve in both the training and validation sets were employed to assess the predictive performance of the prediction model (16). The threshold applied to the validation set was determined by calculating the maximum Youden index from the training set ROC curve (17). In the PR curve, the validation set threshold was determined by calculating the maximum F1 score from the training set PR curve (18). Furthermore, to comprehensively evaluate the predictive accuracy and clinical utility of the model, a calibration curve was used to assess model fit, and decision curve analysis (DCA) was conducted to evaluate the net benefit for patients across various threshold probabilities (19, 20).

### 2.6 Statistical analysis

Statistical analysis was performed using R software (version 4.3.2). The “stats” package was used to construct the logistic regression model, the “rms” package was used to construct the nomogram and calibration curve, the “pROC” package was used to generate the ROC curve, the “PRROC” package was used to generate the PR curve, and the “rmda” package was used to construct the DCA. A normality test was conducted on each set of measured data. If the data followed a normal distribution, the independent-samples t test was used; otherwise, the Mann-Whitney U test was used. The cutoff for metastatic LN diameter was determined based on the maximum Youden index from the ROC curve. The chi-square test was used for the comparison of categorical data. All tests were two-sided, and a p value less than 0.05 was considered to indicate statistical significance.

## 3 Results

### 3.1 Patient characteristics

A total of 198 patients were included in the study (**Table 1**). Postoperative pathological results confirmed that 49 patients were diagnosed with HAE with hepatic hilar LN metastasis (LNM) and 149 patients had no LN metastasis (NLNM) in the hepatic hilar. The ICC for all characteristic features of AE metastatic LNs on CT scans exceeded 0.75 (range: 0.869–1). Compared with patients with NLNM, patients with LNM had significantly larger LN diameters (*P* < 0.001). Sand-like calcification was found in 22.45% of the patients in the LNM group (*P <* 0.001). Nodular calcification was detected in 34.69% of the patients in the LNM group and in 6.04% of the patients in the NLNM group (*P <* 0.001). Hypointense foci areas were detected in 51.02% of the patients in the LNM group and 18.12% of the patients in the NLNM group (*P <* 0.001), and nonenhancement areas were detected in 59.18% of the patients in the LNM group and 20.81% of the patients in the NLNM group (*P <* 0.001). No significant differences in sex, age, body mass index, primary symptoms, location or size of liver lesions, PNM stage, or number of LNs were detected between the two groups (P > 0.05).

**Table 1:** The comparison between the different group of patients.

Variables	No lymph node metastasis N=149	Lymph node metastasis N=49	T value/χ^2^	*P*-value
Gender:			1.333	0.248
Female	77 (51.68%)	29 (59.18%)		
Male	72 (48.32%)	20 (40.82%)		
Age (mean ± SD, yr)	33.9 ± 14.8	35.1 ± 12.7	-0.557	0.583
Body mass index (mean ± SD, kg/m2)	22.3 ± 3.5	21.5 ± 4.4	1.158	0.259
The primary symptom			11.938	0.102
Abdominal pain	20 (13.42%)	9 (18.37%)		
Weight loss	5 (3.36%)	1 (2.04%)		
Jaundice	20 (13.42%)	2 (4.08%)		
Abdominal pain+Weight loss	35 (23.49%)	10 (20.41%)		
Abdominal pain+Jaundice	15 (10.07%)	3 (6.12%)		
Jaundice+Weight loss	13 (8.72%)	12 (24.49%)		
Abdominal pain+Weight loss+Jaundice	19 (12.75%)	6 (12.24%)		
Asymptomatic	22 (14.77%)	6 (12.24%)		
Location of lesions:			3.655	0.1608
Left	47 (31.54%)	9 (18.37%)		
Right	60 (40.27%)	26 (53.06%)		
Both	42 (28.19%)	14 (28.57%)		
Lesion size (mean ± SD, cm2)	98.3 ± 65.7	106.6 ± 59.2	-0.895	0.381
PNM staging:			2.616	0.455
Stage 1 (P1N0M0)	0 (0.00%)	0 (0.00%)		
Stage 2 (P2N0M0)	5 (3.36%)	0 (0.00%)		
Stage 3a (P3N0M0)	23 (15.44%)	6 (12.24%)		
Stage 3b (P1-3N1M0)(P4N0M0)	85 (57.05%)	24 (48.98%)		
Stage 4 (P4N1M0)(P1-4N0-1M1)	36 (24.16%)	19 (38.78%)		
Lymph node diameter (mean ± SD, mm)	13.2 ± 6.20	28.8 ± 14.0	-22.203	<0.001
Number of lymph nodes			0.933	0.3341
1	112 (75.17%)	17 (34.69%)		
≥2	37 (24.83%)	32 (65.31%)		
CT finding of lymph node				
Sand-like calcification	0 (0.00%)	11 (22.45%)	31.267	<0.001
Nodular calcification	9 (6.04%)	17 (34.69%)	24.088	<0.001
Hypointense foci	27 (18.12%)	25 (51.02%)	4.299	<0.001
Non-enhancement	31 (20.81%)	29 (59.18%)	23.936	<0.001
Other lymph node location			6.332	0.387
Posterior pancreatic head lymph nodes	5 (3.36%)	4 (8.16%)		
Para-common hepatic artery lymph nodes	2 (1.34%)	2 (4.08%)		
Para-aorta abdominalis lymph nodes	3 (2.01%)	1 (2.04%)		
Para-diaphragmatic lymph nodes	1 (0.67%)	0 (0.00%)		
Celiac trunk lymph nodes	2 (1.34%)	1 (2.04%)		
Gastric lesser curvature lymph nodes	0 (0.00%)	1 (2.04%)		

There were 40 patients with hepatic hilar LN metastasis only and nine patients with multigroup LN metastasis. There might have been metastases in five other LN groups, including four cases of posterior pancreatic head LNs, two cases of para-common hepatic artery LNs, one case of para-aorta abdominalis LNs, one case of celiac trunk LNs, and one case of gastric lesser curvature LNs. Five other reactive hyperplasia LN sites were identified, including five cases of posterior pancreatic head LN, two cases of para-common hepatic artery LNs, three cases of para-aorta abdominalis LNs, one case of para-diaphragmatic LNs, and two cases of celiac trunk LNs. The chi-square test indicated that there was no significant difference in the incidence of metastatic and reactive hyperplastic LNs at different sites (*P =* 0.387).

### 3.2 Postoperative pathological results of hepatic hilar LNs

During surgery, 491 LNs were dissected, including 114 metastatic and 377 reactive hyperplastic LNs (**Table 2**). Abdominal CT was the most common preoperative diagnostic method for HAE, and all HAE cases were accurately diagnosed. However, CT identified only 80.27% of reactive hyperplastic LNs and 40.23% of metastatic LNs. Importantly, among the 110 LNs deemed normal by CT, 19.09% were found to be metastatic LNs based on postoperative pathological results.

**Table 2:** CT diagnostic accuracy of metastatic LNs in HAE.

Postoperative Pathology preoperative CT scan	No Lymph node metastasis	Lymph node metastasis
Reactive hyperplastic lymph nodes	236 (80.27%)	58 (19.73%)
Metastatic lymph nodes	52 (59.77%)	35 (40.23%)
Normal lymph nodes	89 (80.91%)	21 (19.09%)
Total	377 (76.78%)	114 (23.22%)

### 3.3 Postoperative complications

All patients completed surgery without severe intraoperative complications. There were no significant differences in time or bleeding volume during the operation or postoperative length of stay between the two groups (P > 0.05). All patients experienced Clavien-Dindo complications during their postoperative stay, with no significant differences in complication grades between the groups (P > 0.05). Four patients died from severe postoperative complications following autologous liver transplantation: three from liver failure at 4, 6, and 11 months postsurgery and one from infection-induced multiple organ failure at 7 months postsurgery (**Table 3**).

**Table 3:** The operative outcomes of this study.

Variables	No lymph node metastasis N=149	Lymph node metastasis N=49	T value/χ2	*P*-value
Operation time (mean ± SD, minute)	5.2 ± 1.9	5.6 ± 1.8	-1.331	0.187
bleeding volume during operation (mean ± SD, mm3)	1128.3 ± 363.4	1237.1 ± 409.3	-1.657	0.102
postoperative length of stay (mean ± SD, day)	30.2 ± 18.5	33.6 ± 15.9	-1.245	0.215
Clavien-Dindo complications			4.574	0.334
I	103 (69.13%)	29 (59.18%)		
II	23 (15.44%)	13 (26.53%)		
III	11 (7.38%)	5 (10.20%)		
IV	9 (6.04%)	1 (2.04%)		
V	3 (2.01%)	1 (2.04%)		
Recurrence	4 (3.28%)	2 (4.55%)		0.921 (Cox regression)
Follow-Up Duration in Month (range)	29 (3-112)	40.5 (6-115)	1.81	0.072

### 3.4 Follow-up results

**Figure 3:**
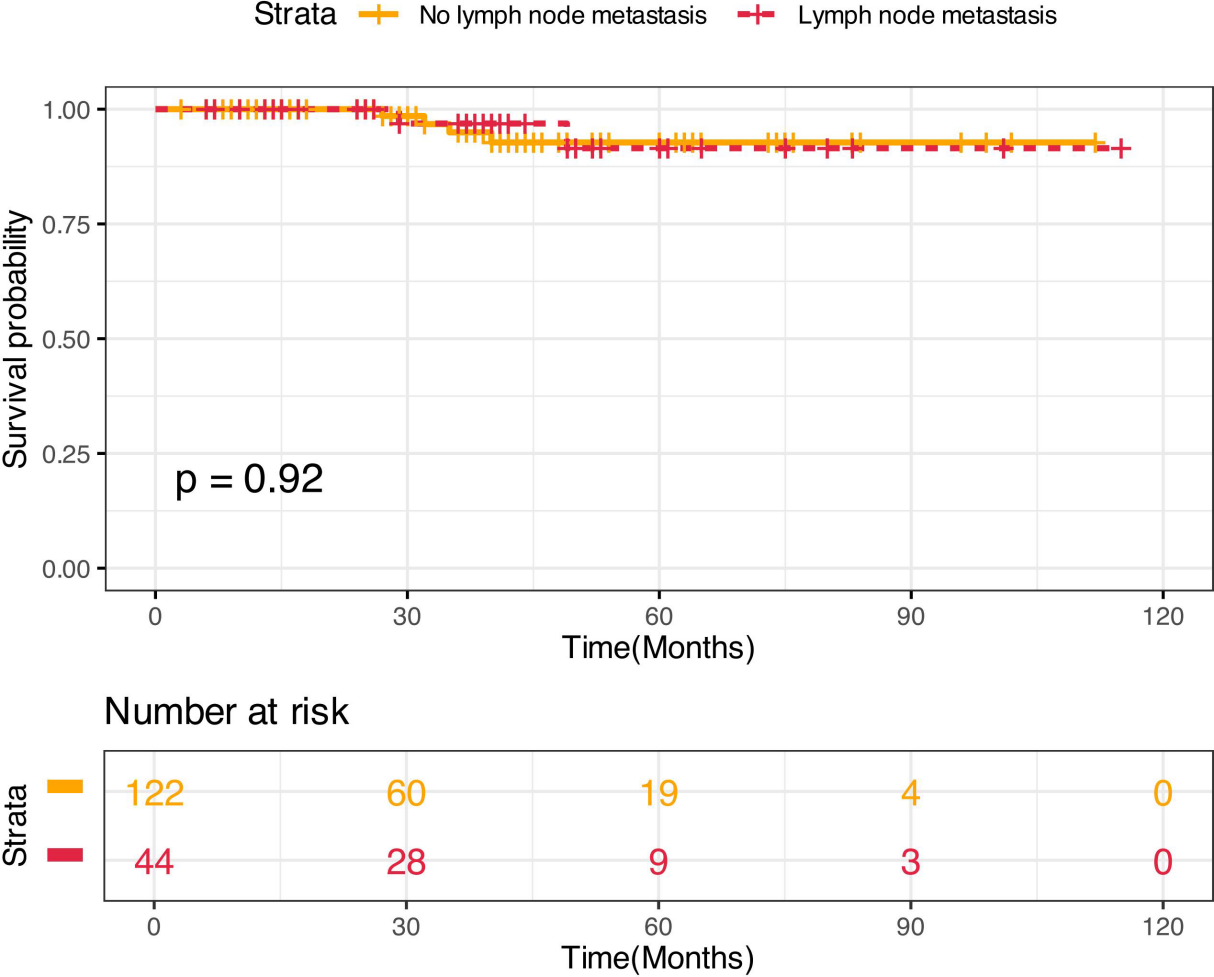
Kaplan-Meier curves comparing DFS between HAE patients with LN metastasis and those with reactive hyperplastic LNs.

The median follow-up was 40.5 months for patients with LNM and 29 months for patients with NLNM. Six patients experienced recurrence (**Table 3**). Among NLNM patients, recurrences occurred at 27, 32, 35, and 40 months postsurgery. For patients with LNM, recurrence occurred at 29 and 49 months. The Kaplan-Meier analysis revealed no significant difference in DFS between the NLNM and LNM groups (log-rank *P* = 0.920) (**Figure 3**). All recurrences were located at the liver resection margins, with one LNM patient developing new hepatic lesions. No extrahepatic lesions were detected in any recurrent cases.

### 3.5 Model development

**Figure 4:**
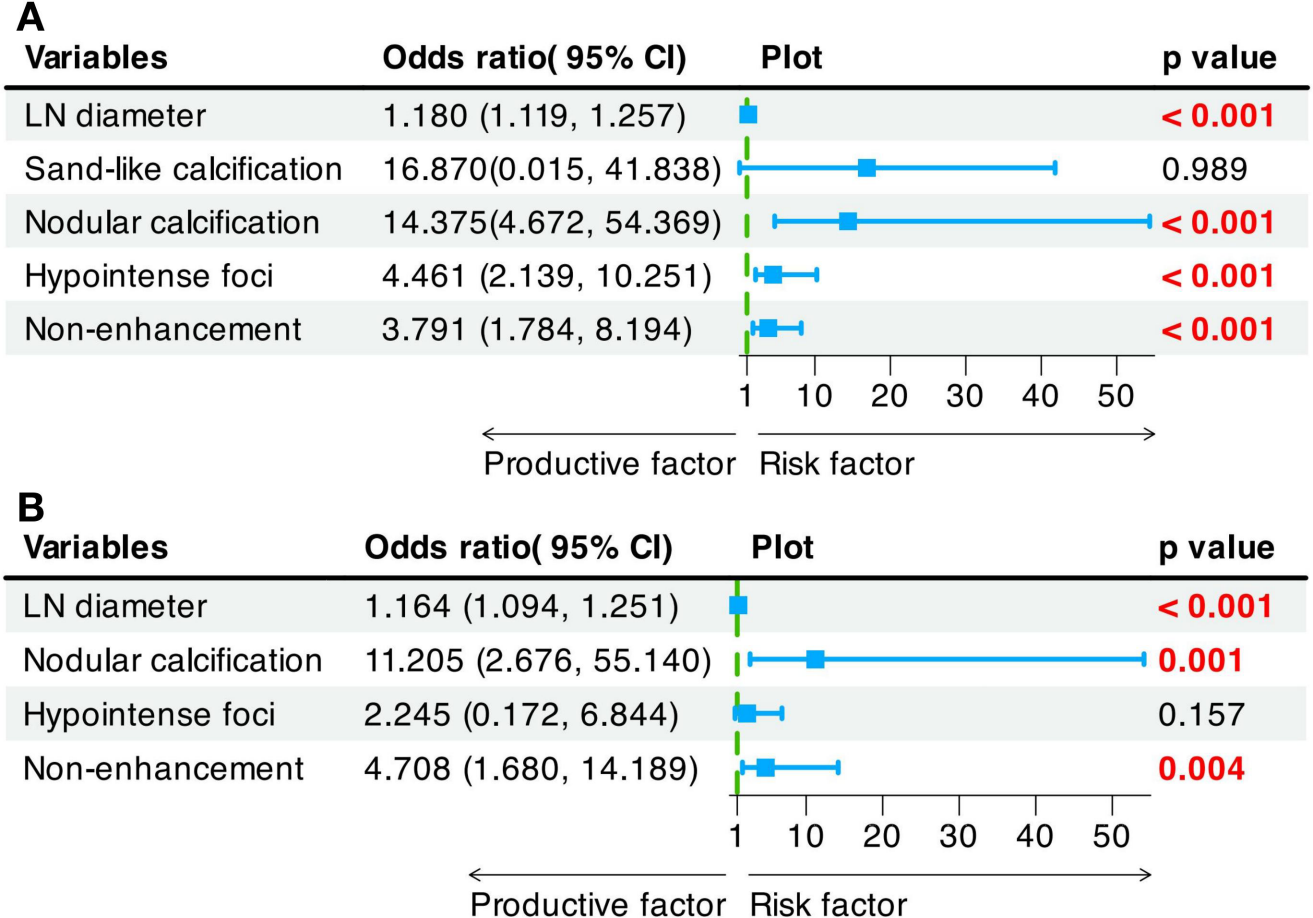
Univariate **(A)** and multivariate **(B)** Logistic regression analysis of LN metastasis in HAE.

**Figure 5:**
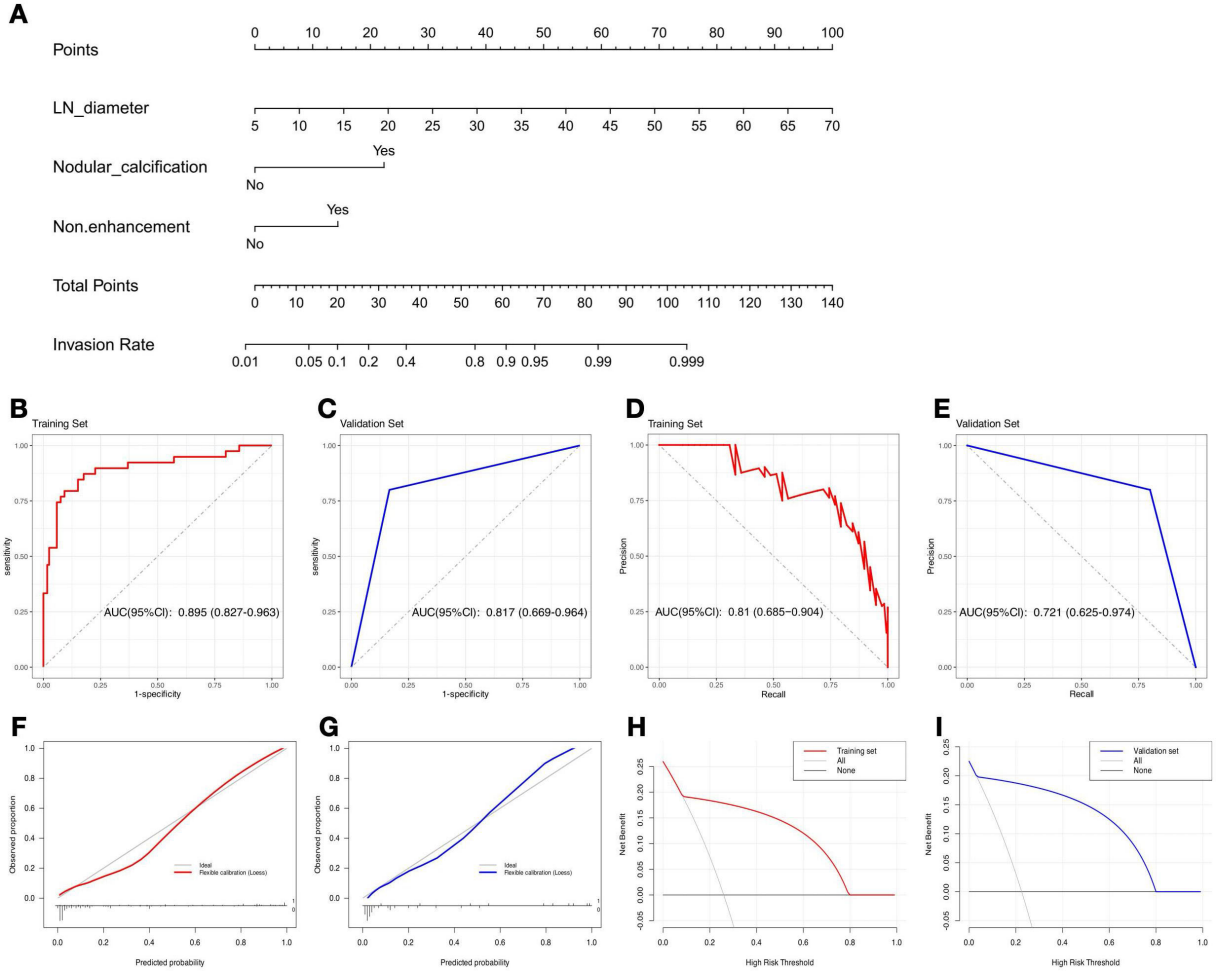
Nomogram and the predictive efficacy of the model. **(A)** The nomogram was developed with the training set. ROC curves of the training set **(B)** and validation set **(C)**. PR curves of the training set **(D)** and validation set **(E)**. Model calibration curves for training set **(F)** and validation set **(G)**. DCA of training set **(H)** and validation set **(I)**.

Univariate logistic regression revealed that LN diameter (OR = 1.180 [95% CI: 1.119 - 1.257], *P* < 0.001), nodular calcification (OR = 14.375 [95% CI: 4.672 - 54.369], *P* < 0.001), hypointense foci (OR = 4.461 [95% CI: 2.139 - 10.251], *P <* 0.001), and nonenhancement (OR = 3.791 [95% CI: 1.784 - 8.194], *P* < 0.001) were significant risk factors for hepatic hilar LN metastasis. Multivariate logistic regression analysis further revealed LN diameter (OR = 1.164 [95% CI: 1.094 - 1.251], *P* < 0.001), nodular calcification (OR = 11.205 [95% CI: 2.676 - 55.140], *P* = 0.001), and nonenhancement (OR = 4.708 [95% CI: 1.680 - 14.189], *P* = 0.004) as independent predictors of hepatic hilar LN metastasis in HAE patients (**Figure 4**). By incorporating these independent predictors into a logistic regression model, a predictive model for hepatic hilar LN metastasis in HAE patients was developed: Logit(*P*) = 15.177 + 0.165 × LN diameter + 2.404 × Nodular calcification + 1.544 × Nonenhancement (**Figure 5A**). LN diameter is entered as a continuous variable (unit: mm); both nodular calcification and nonenhancement are binary variables, coded as 1 when present and 0 otherwise. The scatter plots of the predicted probabilities for each patient in the training and validation sets are shown in **Supplementary Figure 2**.

### 3.6 Predictive performance of the model

The VIF values for all three variables in the model were less than 5, suggesting negligible multicollinearity, which had a minor effect on the model (LN diameter: 1.196; nodular calcification: 1.157; nonenhancement: 1.036). The E values of the LN diameter, nodular calcification, and nonenhancement were 1.641 (1.487 for lower 95% CI), 7.283 (2.958 for lower 95% CI), and 28.241 (8.814 for lower 95% CI), respectively, indicating that a potential factor or combination of factors would need to have a large OR to weaken the association between the predictors and LN metastasis.

A clinical application example of the model is shown in **Supplementary Figure 1**, which successfully determined the status of suspicious LNs and guided the operation methods. The model’s predictive performance is summarized in **Table 4**. The training set results (sensitivity = 0.6, specificity = 0.941) align closely with those of the validation set (sensitivity = 0.8, specificity = 0.867). Analysis of the ROC and PR curves confirmed the robust classification capability of the model. The AUC-ROC values were 0.895 (95% CI: 0.827-0.963) and 0.817 (95% CI: 0.669-0.964) in the training set and validation set, respectively (**Figures 4B, C**). The AUC-PR values were 0.81 (95% CI: 0.685-0.904) and 0.721 (95% CI: 0.625-0.974) in the training set and validation set, respectively (**Figures 4D, E**). In both sets, the calibration curves exhibited a close match between the predicted probabilities and actual probabilities, demonstrating good discrimination and calibration capabilities (**Figures 4F, G**). The DCA results revealed that the model’s performance was consistently greater than the extreme curve throughout the entire range, further indicating its good clinical applicability (**Figures 4H, I**). Overall, our model may have strong predictive performance and can accurately distinguish HAE hepatic hilar LN metastasis preoperatively.

**Table 4:** Prediction performance of the model.

Parameter	Training set	Validation set
Cutoff	0.354	0.354
Recall	0.590	0.800
Precision	0.767	0.667
Sensitivity	0.600	0.800
Specificity	0.941	0.867
Accuracy	0.854	0.850
F1	0.667	0.727
Brier	0.095	0.081
AUC for the ROC curve (95% CI)	0.895 (0.827-0.963)	0.817 (0.669-0.964)
AUC for the PR curve (95% CI)	0.810 (0.685-0.904)	0.721 (0.625-0.974)

## 4 Discussion

The liver is the organ most frequently affected by EM in humans, accounting for approximately 96.8% of all AE cases (21). The disease can metastasize to surrounding tissues and distant organs, such as the lungs, brain, and kidneys, via direct invasion, vascular spread, or lymphatic dissemination (22, 23). In clinical practice, HAE with regional LN involvement is not uncommon; approximately 5.7% to 8.83% of patients with HAE present with LN metastases (24, 25). In our center, the incidence of LN metastasis among patients with HAE over the past nine years was 9.57%, which is consistent with the findings of previous reports. Residual infected LNs may act as a source of recurrence if not thoroughly resected during surgery. Studies report that recurrence rates following radical hepatectomy alone range from 2% to 16% (11, 26–28). However, the recurrence rate decreases significantly to 2%–8% when concomitant LN dissection is performed (25, 29). Although the mechanisms underlying LN metastasis and its role in disease recurrence are not fully understood, evidence suggests that a proportion of recurrences may be attributable to undetected metastatic LNs (30). Thus, prompt identification and treatment of infected LNs are essential for improving their prognosis.

Preoperative abdominal ultrasound, MRI, and CT demonstrated a diagnostic accuracy of 100% for HAE, yet their sensitivities for detecting LN metastases were considerably lower, at 16.4%, 18.2%, and 30.9%, respectively (25, 31). Although CT exhibited relatively better performance, only larger LNs with typical morphological features could be detected at an early stage. Moreover, among those LNs with imaging characteristics, 16% to 36% were ultimately confirmed as reactive hyperplasia (8, 24). Therefore, the distinction of metastatic LNs is important for personalized treatment planning. However, research in this area remains limited, and only Zhou et al. have developed a CT-based radiomics model for predicting metastatic LNs in enlarged hepatic hilar LNs (AUC = 0.928) (8). Nevertheless, its reliance on specialized software may hinder its broader clinical application. The parameters selected for the model in this study are directly assessable CT image features. Compared with radiomics models, its primary advantage lies in its excellent clinical accessibility, providing primary hospitals with a convenient and practical risk assessment tool. Retrospective analysis of patients at our institution revealed several CT imaging characteristics associated with metastatic LNs, including LN diameter, sand-like calcifications, nodular calcification, hypointense foci and nonenhancement areas on contrast-enhanced scans. Among these factors, only the LN diameter, nodular calcification, and nonenhancement areas were identified as independent predictive factors. Although sand-like calcification is a typical CT feature of metastatic LNs and is observed significantly more frequently than in reactive hyperplastic LNs, it did not demonstrate statistical significance in the logistic regression model. This may be attributed to its low incidence (5.3%) in metastatic LNs, suggesting limited clinical utility even if a statistical association were present. A marker with low sensitivity is unlikely to retain statistical significance in multivariate analyses. From a mechanistic perspective, sand-like calcifications may reflect a chronic inflammatory process rather than a metastasis-specific change. Therefore, it serves as an indicative feature for LN metastasis in patients with HAE rather than a reliable predictor. In contrast, nodular calcification showed stronger independent predictive value, likely because of its higher specificity in HAE, as it represents calcified remnants of degenerated parasites and associated inflammatory responses. Additionally, nodular calcification reached statistical significance because of an adequate sample size and the absence of multicollinearity with other features, enabling it to independently distinguish metastatic from reactive hyperplastic LNs. The nonenhancement areas on contrast−enhanced imaging may be attributed to both the biological traits of the parasite and the immune response of the host. First, infiltrative growth is a hallmark of HAE, resulting in avascular structures within metastatic LNs (32). EM further suppresses peri−lesional angiogenesis and promotes fibrosis by stimulating immune cells to release immunosuppressive factors such as TGF−β1 and NF−κβ (33, 34), thereby reducing local blood perfusion. Additionally, calcification and liquefactive necrosis within metastatic LNs can hinder contrast uptake. Together, these factors lead to failure of contrast agent penetration, manifesting as the characteristic “avascular area” in metastatic LNs. In this study, the cutoff for the diameter of metastatic LNs was 20.26 mm, which is significantly higher than that of reactive hyperplastic LNs. Reactive hyperplasia occurs when inflammatory mediators and small EM particles drain via the lymphatic system to regional LNs, eliciting varying degrees of immune response (35). This process leads to localized fibrosis, chronic inflammation, edema, and hyperplasia of the LNs, eventually resulting in characteristic granulomatous changes. Such reactive hyperplasia is generally self−limiting and does not typically cause excessive enlargement, with diameters typically ranging from 1–2 cm (36). In contrast, HAE is a parasitic disease exhibiting malignant tumor characteristics. The enlargement of metastatic LNs in HAE is attributed not only to immune reactions but also to the infiltrative and destructive growth of the EM (37). This disrupts normal LN architecture and contributes significantly to LN enlargement. Additionally, inflammatory factors such as IL-6 and TNF-α secreted by HAE lesions continuously stimulate lymphatic tissues (38, 39), further promoting LN enlargement. In contrast, reactive hyperplasia is typically triggered by transient immune stimulation, which makes it self-limiting.

AUC and PR curves were used complementarily in this study to assess model performance. The ROC curve has the advantage of remaining stable even when the proportion of positive and negative samples in the dataset is unbalanced and is suitable for evaluating the global performance of the model. However, this study had a typical unbalanced dataset, with a relatively low proportion of positive cases (24.75%), and the AUC-ROC may have been magnified because of the dilution of the false-positive rate (FPR) by many negative samples. In contrast, the PR curves focus on precision and recall for positive cases, with the advantage that they are more sensitive to data with high class imbalance, and direct exposure models have insufficient recognition of positive cases if the AUC-PR is low (40). Our model had good AUC-ROC and AUC-PR, but the former was significantly better, suggesting that there may be a risk of missed or misdiagnosed metastatic LN cases. This may be related to the lack of absolute specificity of the model features, which need to be improved by feature optimization or a sampling strategy.

There is no clear international standard, so different centers have different treatment strategies for metastatic LNs in HAE. There are very few papers describing this topic worldwide. Aimaitijiang et al. reported that among 55 patients with HAE LN metastases observed for more than a decade, only one (1.8%) experienced disease recurrence in the liver eight years post-operatively (25). A retrospective single-center study that enrolled 127 HAE patients suspected of having LN metastasis who underwent radical hepatectomy with regional LN dissection over a 9-year period reported recurrence in eight patients (7.86%) without LN metastasis and in two patients (8.33%) with LN metastasis (*P* = 0.525) (24). Andreas et al. followed a cohort of 109 patients with HAE who underwent radical surgery. Of these, 43 were found to have enlarged LNs, but they underwent only targeted dissection of the enlarged LNs rather than regional LN dissection. During the 16-year follow-up period, recurrence was observed in two (4.65%) patients with reactive hyperplastic LNs and eight (12.12%) patients without any LN resection (29). Notably, none of the seven patients with metastatic LNs experienced disease recurrence during the study period.

Our institution has personalized strategies for managing LNs on the basis of CT scans and intraoperative exploration. Given that most LN metastases occur in the hepatic hilar LNs near the liver, detecting one metastatic LN with imaging features suggests that additional LNs may be in a latent stage of infection. In infected LNs, when abnormal components such as EM and granulation tissue completely block the lymphatic vessels to the downstream LNs, germinal layer cells may migrate into the liver tissue upstream against the flow of the lymphatic fluid, causing retrograde lymphatic metastasis (7, 41, 42). Next, as lymph fluid eventually drains into the bloodstream, lymphatic metastasis can progress to hematogenous spread to distant organs (43). Consequently, hepatic hilar LN dissection is necessary once typical metastatic hepatic hilar LNs appear. The occurrence of LN metastases at other sites is considerably lower than that of hepatic hilar LNs, and the increased distance from the liver reduces the likelihood of cells in the germinal layer migrating back to the liver. In light of surgical safety and patient benefits, we only remove potentially metastatic LNs at sites other than hilar LNs rather than perform regional LN dissection.

The incidence of severe complications (Clavien-Dindo grade ≥ III) was similar between patients with LMN (14.3%) and those with NLMN (15.4%) (*P* = 0.334). Severe complications were primarily associated with factors such as extensive surgical trauma and preoperative multiorgan dysfunction rather than with LN dissection. Recurrence occurred primarily in the liver, likely because of incomplete resection of lesions rather than extrahepatic tissues. Following standardized treatment, patients with HAE exhibit comparable DFS regardless of LN metastasis status. This observation does not negate the risks associated with LN metastasis; rather, it emphasizes that standardized surgical intervention can enable such patients to achieve long-term outcomes similar to those without metastasis.

Accurate preoperative identification of LN metastasis is crucial for determining the extent of LN dissection and enhancing surgical safety and efficacy. At our institution, the detection rate of metastatic LNs via CT scan was 50.88%. Among the 114 pathologically confirmed metastatic LNs, 35 (40.23%) were incorrectly identified as reactive hyperplastic LNs, and 21 (19.09%) were misclassified as normal LNs. Current imaging techniques struggle to accurately identify potential metastatic LNs that lack distinct imaging features. Many scholars believe that potential metastatic LNs may be the source of persistent infection, suggesting that the surgical scope should be extended to regional LNs to reduce the risk of recurrence. Klaus et al. reported a case of HAE with LN metastasis, illustrating pathological progression from the liver to regional LNs. Solange et al. conducted a 20-year follow-up of five patients with advanced HAE who underwent liver transplantation without LN dissection. Eventually, recurrence occurred in the grafts and LNs, indicating that residual false-negative LN metastases might be the cause of recurrence (30). Identifying new risk factors for LN metastasis is crucial to avoid both undertreatment and overtreatment.

Our study has several limitations. Imaging follow-up for more than 10 years after HAE treatment can determine whether a cure has been achieved (44, 45), the follow-up period of 9 years in this study was obviously insufficient, and longer-term observation is needed. Despite rigorous data processing and validation, the restricted sample size could have introduced bias, impacting the generalizability of the results. Future research should encompass multicenter prospective studies to confirm the model’s predictive accuracy and assess the effectiveness of surgical interventions. Additionally, while this study exclusively utilized contrast-enhanced CT scans, future research should integrate multimodal data such as MRI and ultrasound to acquire more reliable predictive variables.

## 5 Conclusion

This study, which is based on the largest current cohort of patients with HAE, identified LN diameter, nodular calcification, and nonenhancement on contrast-enhanced CT scans as independent predictors of hepatic hilar LN metastasis in patients with HAE. A model was successfully developed to predict the risk of LN metastasis and aid clinicians in decision-making. Furthermore, the 9-year follow-up data from our institution support that radical hepatectomy combined with SHD-TR is safe and effective. These findings are anticipated to guide future management strategies for patients with HAE with LN metastasis.

## Data Availability

The raw data supporting the conclusions of this article will be made available by the authors, without undue reservation.
