# Supplementary material for: Diagnosis and treatment of hepatic hilar lymph node metastasis in hepatic alveolar echinococcosis patients: a real-world single-center experience
Source: Front Oncol. 2026 Jan 21;15:1708936. doi: 10.3389/fonc.2025.1708936 (PMC12867826; doi:10.3389/fonc.2025.1708936)
Supplement: Supplementary file 1 [file Image1.pdf]

Supplementary Material

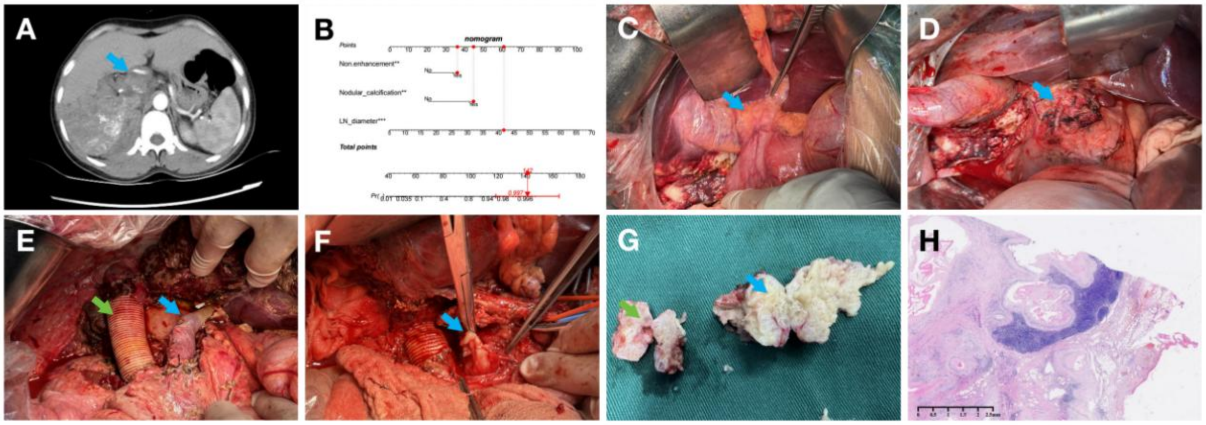

Supplementary Figure 1 A 34-year-old female was assessed using a model to predict HAE LNs metastasis. (A) A preoperative CT scan indicated suspected HAE with hepatic hilar LN metastasis (blue arrow). (B) Contrast-enhanced CT scan showed non-enhancement = "1", nodular calcification = "1", and LN diameter was 42 mm. The total score was 142, which corresponds to a risk of LN metastasis of approximately 0.997. Due to a high score suggested a significant risk of LN metastasis, a radical hepatectomy with hepatic hilar LN dissection was scheduled after communicating with the patient. (C) Enlarged and fused hepatic hilar LNs were identified intraoperatively, consistent with the preoperative CT scan (blue arrow). (D) Upon dissection of the Glisson's sheath, enlarged and fused hepatic hilar LNs were found surrounding the hepatic artery and closely adhering to the common hepatic duct and portal vein (blue arrow). (E) The hepatic hilar structure after hepatic hilar LN dissection (blue arrow) and the reconstructed inferior vena cava (green arrow). (F) Systematic LN exploration revealed an enlarged and hard para-aorta abdominalis LN, which was considered metastatic and were resected (blue arrow). (G) Pathological examination of the suspicious hepatic hilar LN (blue arrow) and para-aorta abdominalis LN (green arrow) confirmed the presence of AE lesions. (H) Hematoxylin-eosin staining at 1× magnification demonstrated metastatic AE in the suspicious hepatic hilar LN. (Note: Metastatic LNs invaded the patient's biliary tract and caused stenosis, so cholangiojejunostomy was performed later. The metastatic LNs also affected the proper and common hepatic arteries. The proper hepatic artery and common hepatic artery of the patient were invaded by metastatic LNs. Due to the presence of an accessory left hepatic artery, reconstruction of the proper and left hepatic arteries was not performed following hilar LN dissection. The surgery was successful, and the patient showed a good recovery, with no recurrence detected four years post-operation.) LN: Lymph node; HAE: hepatic alveolar echinococcosis.
