# Supplementary material for: Diagnosis and treatment of hepatic hilar lymph node metastasis in hepatic alveolar echinococcosis patients: a real-world single-center experience
Source: Front Oncol. 2026 Jan 21;15:1708936. doi: 10.3389/fonc.2025.1708936 (PMC12867826; doi:10.3389/fonc.2025.1708936)
Supplement: Supplementary file 2 [file Image2.pdf]

Supplementary Material

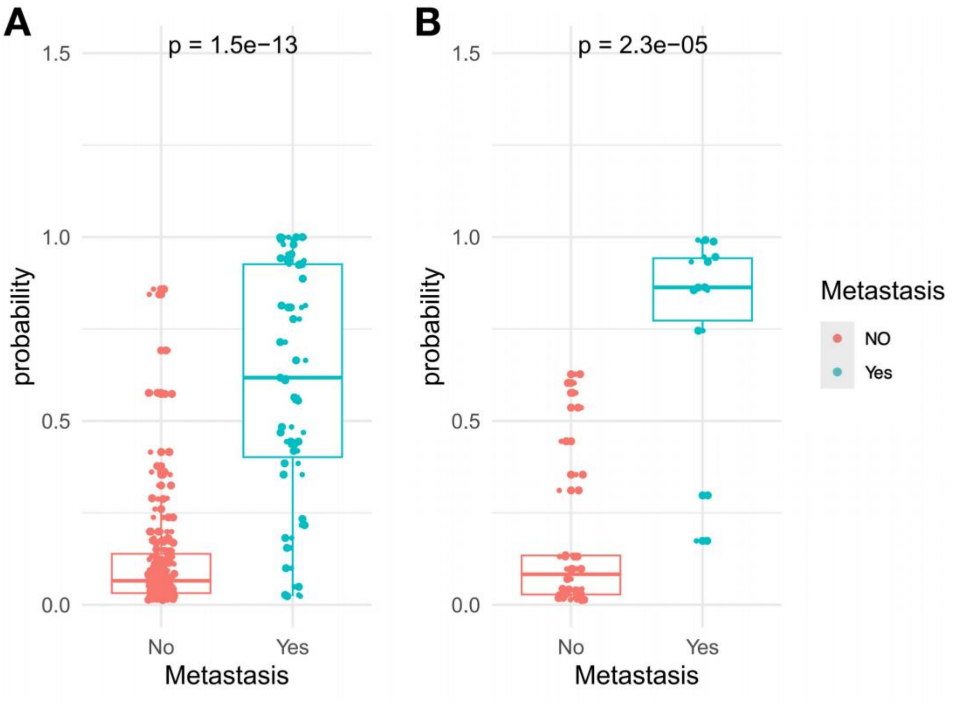

Supplementary Figure 2 The scatter plots of the predicted probabilities for each patient in the training and validation sets.
